# Optimization of Flavonoid Extraction from Red and Brown Rice Bran and Evaluation of the Antioxidant Properties

**DOI:** 10.3390/molecules23081863

**Published:** 2018-07-26

**Authors:** Ali Ghasemzadeh, Ali Baghdadi, Hawa Z. E. Jaafar, Mallappa Kumara Swamy, Puteri Edaroyati Megat Wahab

**Affiliations:** Department of Crop Science, Faculty of Agriculture, Universiti Putra Malaysia, Serdang 43400, Selangor, Malaysia; ali_baghdadi@yahoo.com or ali_baghdadi@upm.edu.my (A.B.); hawazej@upm.edu.my (H.Z.E.J.); swamy.bio@gmail.com (M.K.S.); putri@upm.edu.my (P.E.M.W.)

**Keywords:** central composite design, response surface methodology, rice bran, flavonoids, antioxidant activity

## Abstract

Recently, the quality-by-design concept has been widely implemented in the optimization of pharmaceutical processes to improve batch-to-batch consistency. As flavonoid compounds in pigmented rice bran may provide natural antioxidants, extraction of flavonoid components from red and brown rice bran was optimized using central composite design (CCD) and response surface methodology (RSM). Among the solvents tested, ethanol was most efficient for extracting flavonoids from rice bran. The examined parameters were temperature, solvent percentage, extraction time, and solvent-to-solid ratio. The highest total flavonoid content (TFC) in red rice bran was predicted as 958.14 mg quercetin equivalents (QE)/100 g dry matter (DM) at 58.5 °C, 71.5% (*v*/*v*), 36.2 min, and 7.94 mL/g, respectively, whereas the highest TFC in brown rice bran was predicted as 782.52 mg QE/100 g DM at 56.7 °C, 74.4% (*v*/*v*), 36.9 min, and 7.18 mL/g, respectively. Verification experiment results under these optimized conditions showed that the TFC values for red and brown rice bran were 962.38 and 788.21 mg QE/100 g DM, respectively. No significant differences were observed between the predicted and experimental TFC values, indicating that the developed models are accurate. Analysis of the extracts showed that apigenin and *p*-coumaric acid are abundant in red and brown rice bran. Further, red rice bran with its higher flavonoid content exhibited higher nitric oxide and 2,2-diphenyl-1-picrylhydrazyl scavenging activities (EC_50_ values of 41.3 and 33.6 μg/mL, respectively) than brown rice bran. In this study, an extraction process for flavonoid compounds from red and brown rice bran was successfully optimized. The accuracy of the developed models indicated that the approach is applicable to larger-scale extraction processes.

## 1. Introduction

In recent years, there has been increased interest in natural sources that could provide active components to prevent the impact of free radicals on cells [1,2]. For this reason, studies on natural antioxidants have increased considerably. Rice bran is the most abundant and valuable byproduct of the rice milling process [3]. Phytochemical analysis of rice bran has shown that rice bran is rich in γ-oryzanol (mixture of ferulic acid esters of sterols and triterpene alcohols) [4], phytic acid, tocopherols, tocotrienols, carotenoids, aminobutyric acid, octacosanol, squalene, unsaturated fatty acids, phytosterols, flavonoids, and phenolic compounds [5]. Pigments in so.me rice varieties appear in the rice bran layer, resulting in pigmented rice of different colors. The color of the pigments depends on the presence of various compounds, such as anthocyanin and proanthocyanidin, based on which pigmented rice is classified [6]. In particular, several types of polyphenolic compounds have been reported in pigmented rice bran, especially brown and red rice bran [7]. The pharmaceutical quality of pigmented rice bran is associated with the type and content of specific phytochemicals, such as flavonoids [7,8]. Hence, the recovery of flavonoids from pigmented rice bran may by commercially beneficial in terms of natural antioxidant isolation. Extraction processes for secondary metabolites from pigmented rice bran is critical [9], and the polarity and concentration of the extraction solvent have been reported as important factors [10,11]. To extract different types of secondary metabolites from plant sources, various types of solvents, such as methanol, ethanol, and acetone, have been commonly used [10,12,13]. Results of previous studies recommended acetone and ethanol for secondary metabolites extraction from rice bran [14,15]. The use of these organic solvents in extraction processes depends on the plant variety and the targeted compounds [16]. Optimization of secondary metabolites extraction from pigmented rice was reported in some studies [17,18]. However, these techniques require specific equipment (ultrasonic, microwave) to extract secondary metabolites or they did optimization whiteout using of appropriate statistical method (response surface methodology). Finding a simple method with a higher extraction yield, reducing of solvent using, energy using and time could be useful to extract secondary metabolites from the pigmented rice bran on a large scale. However, for large-scale processes, it is essential to optimize the variables that are critical to the extraction process to maximize the yield of the targeted compound. Further useful information and optimal experimental conditions can be achieved by using a good experimental design and a suitable model. Response surface methodology (RSM) was developed to optimize various extraction processes, including the extraction variables such as solvent polarity, extraction time, and temperature [19,20,21]. The various parameters and their interactions can be evaluated efficiently with this data analysis technology, reducing the experimental group number [22,23]. The production of secondary metabolites in the pigmented rice may vary for various reasons, including varietal difference, climate changes, agricultural practices, etc. [24,25,26]. Hence, optimizing an efficient of extraction process is very important for a particular variety. To the best of our knowledge, this is the first report describing optimization of extraction process of secondary metabolites from Iranian pigmented rice bran. Also, developing an efficient and environmental friendly method for the extraction of secondary metabolites from the pigmented rice varieties is necessary.

Therefore, the aim of this study was to investigate and optimize various parameters, such as temperature, extraction time, solvent-to-solid (S/S) ratio, and solvent polarity, for the extraction of flavonoids from red or brown rice bran using central composite design (CCD) and RSM. In addition, individual flavonoids, phenolic acid profiling, and the antioxidant activity of the optimized extracts were evaluated.

## 2. Results and Discussion

### 2.1. Preliminary Studies/Analysis of Single-Factor Method

**Figure 1:**
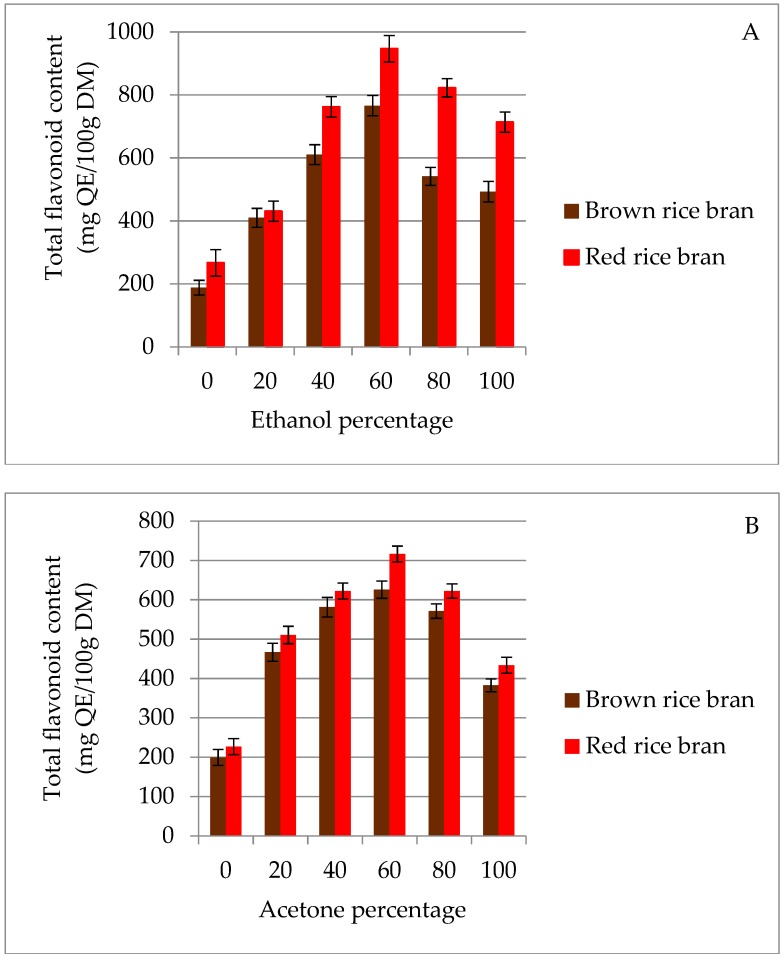
Effect of ethanol (**A**) and acetone (**B**) solvents on the extraction yield of total flavonoid content (TFC) for red and brown rice bran.

In the current study, two solvents, namely acetone and ethanol, with different polarities were used to extract flavonoids from two pigmented (red and brown) rice brans. A significant difference between acetone and ethanol was observed for the extraction of total flavonoids from rice bran (Figure 1). In red and brown rice bran, the highest total flavonoid content (TFC) values (946.4 and 765.8 mg quercetin equivalents (QE)/100 g dry matter (DM), respectively) were observed in 60% (*v*/*v*) ethanolic solution (Figure 1A). This result may be due to the similar polarities of this solution and the flavonoids present in the bran. In contrast, it can be observed from Figure 1B that TFC extraction in acetone was not significantly different for red and brown rice bran at concentrations of 0–40% (*v*/*v*). This behavior may result from the lower polarity of the acetone medium [27], which inhibits the extraction of higher polarity substances from the rice bran and, hence, the TFC value plateaus. The lowest TFC value from both rice brans was observed for 100% water. Moreover, extraction with pure water as a solvent resulted in high impurities, which led to a decrease in flavonoid quantity [28]. The results of this study show that for extraction of flavonoids from red and brown rice bran, ethanol and acetone are more efficient than pure water. Therefore, an extraction solvent of 40–80% (*v*/*v*) ethanol or acetone was chosen in subsequent experiments.

**Figure 2:**
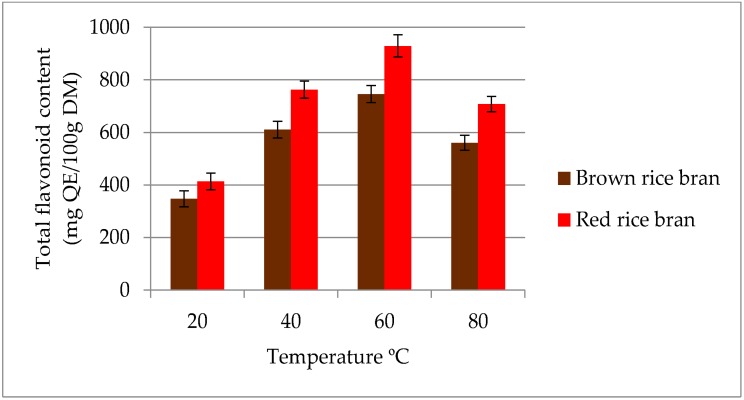
Effect of different temperatures on the extraction yield of total flavonoid content (TFC) for red and brown rice bran.

The influence of temperature on the extraction yield of total flavonoids in ethanolic (50% *v*/*v*) extracts is given in Figure 2. The maximum TFC was obtained at 60 °C for both rice brans. In contrast, the minimum TFC value was observed at 20 °C for both red (412.8 mg QE/100 g DM) and brown rice bran (346.7 mg QE/100 g DM) extracts. An increased temperature during extraction softens plant tissues, thereby weakening phenol–protein and phenol–polysaccharide interactions. Such weakened interactions may enhance the diffusion of secondary metabolites, such as flavonoids, into the extraction media. Moreover, compositional differences between the secondary metabolites in red and brown rice bran could account for the variation with extraction temperature. Oxidation and decomposition of secondary metabolites at higher temperatures results in a reduction of flavonoid compounds in extracts [29,30]. Therefore, an extraction temperature range of 40–80 °C was selected for subsequent experiments.

**Figure 3:**
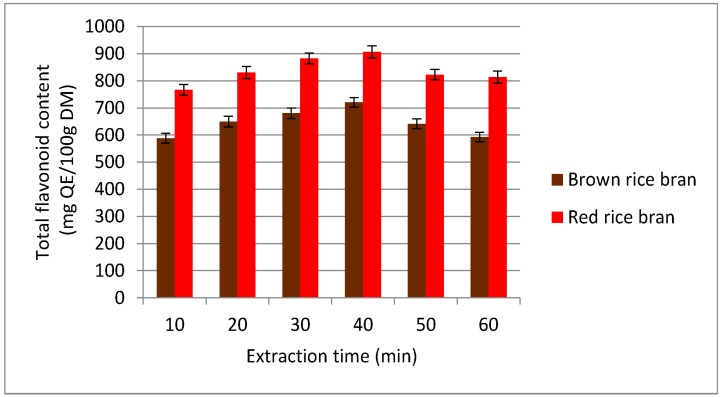
Effect of different extraction times on the extraction yield of total flavonoid content (TFC) for red and brown rice bran.

To economize the secondary metabolite extraction process, reducing/minimizing the extraction time is essential. In the current study, an extraction time range of 10–60 min was tested for recovery of flavonoids (Figure 3). On increasing the extraction time from 10 to 40 min, the TFC value increased significantly. Further increasing the extraction time to 60 min resulted in a decrease of the TFC value. From these results, it was determined that flavonoid extraction from red and brown rice bran is complete within approximately 40 min. The reduction of the secondary metabolite content in the extract at higher temperatures was explained by Fick’s second law [31], which states that the concentrations of compounds in the extract reach a final equilibrium after a certain time and after this time, no further recovery can be achieved [32]. Furthermore, the decrease in the TFC at longer extraction times might be due to changes in the molecular structure or oxidation of flavonoids [33]. Hence, for further experiments, an extraction time of 30–50 min was selected.

**Figure 4:**
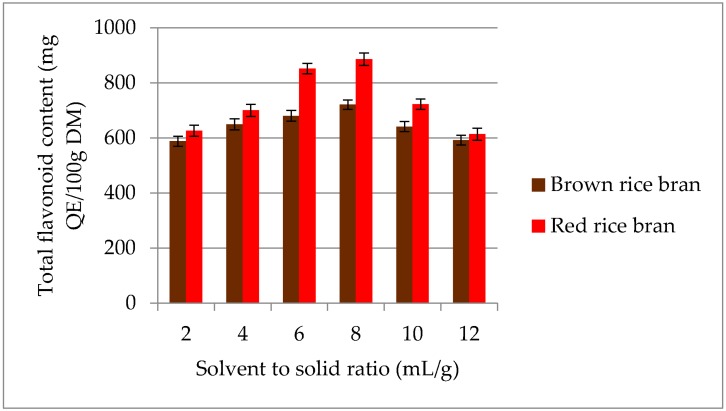
Effect of different S/S ratio on the extraction yield of total flavonoids content (TFC) for red and brown rice bran.

The highest TFC was recorded at an S/S ratio of 8 mL/g for both red (886.4 mg QE/100 g DM) and brown rice bran (720.8 mg QE/100 g DM) (Figure 4). On increasing the S/S ratio from 8 to 12 mL/g, the TFC decreased significantly. The initial increase in the TFC with increasing S/S ratio is correlated with mass-transfer principles. Diffusion of secondary metabolites into the solvent (mass transfer) during the extraction process depends on the concentration gradient between the solid and liquid [34]. However, above a certain S/S ratio, a decrease in the extraction yield was observed. It is possible that larger amounts of liquid do not change the driving force further because the mass-transfer process is highly restricted to the solid interior, thus decreasing total flavonoid recovery [35]. Therefore, in the current study, an S/S ratio of 6–10 mL/g was selected for further experiments.

### 2.2. Model and Response Surface Analysis

The levels of extraction variables (time, temperature, percentage of solvent, and S/S ratio) were selected for the experimental design after the preliminary investigations. Rotatable central composite design (CCD) and RSM were used for regression and response surface analysis.

### 2.3. Model Fitting

Analysis of variance for the experimental results of TFC from red and brown rice bran is shown in Table 1 and Table 2. The linear and corresponding quadratic parameters of extraction temperature (X_1_), ethanol percentage (X_3_), and S/S ratio (X_4_) were found to be significant (*p* < 0.05). The results of the statistical analysis for the interactions of X_1_X_3_, X_1_X_4_, and X_3_X_4_ were also significant (*p* < 0.01). However, the extraction time (X_2_) showed no significant linear, quadratic, or interaction effect. The predicted models developed for flavonoid extraction from red (Y_1_) and brown (Y_2_) rice bran were as follows:

Y_1_ = +745.34 − 62.86X_1_ − 9.35X_2_ + 29.11X_3_ − 18.10X_4_ + 1.48X_1_X_2_ − 30.21X_1_X_3_ + 31.13X_1_X_4_ − 4.17X_2_X_3_ − 3.41X_2_X_4_ + 18.48X_3_X_4_ − 152.50X_1_^2^ − 14.65X_2_^2^ − 58.50X_3_^2^ − 17.90X_4_^2^

Y_2_ = +814.26 − 54.44X_1_ − 9.28X_2_ + 28.59X_3_ − 18.30X_4_ + 1.67X_1_X_2_ − 31.07X_1_X_3_ + 30.85X_1_X_4_ − 3.65X_2_X_3_ − 3.36X_2_X_4_ + 19.04X_3_X_4_ − 153.21X_1_^2^ − 13.93X_2_^2^ − 56.18X_3_^2^ − 18.48X_4_^2^

After three revisions of the regression equation and elimination of non-significant items (*F*-value < *F*-critical value, *p* > 0.05), the predicted models established for flavonoids (Y_1_ and Y_2_) were modified as:

Y_1_ = +745.34 − 62.86X_1_ + 29.11X_3_ − 18.10X_4_ − 30.21X_1_X_3_ + 31.13X_1_X_4_ + 18.48X_3_X_4_ − 152.50X_1_^2^ − 58.50X_3_^2^

Y_2_ = +814.26 − 54.44X_1_ + 28.59X_3_ − 18.30X_4_ − 31.07X_1_X_3_ + 30.85X_1_X_4_ + 19.04X_3_X_4_ − 153.21X_1_^2^ − 56.18X_3_^2^

Based on the elimination consequence, X_2_, X_2_^2^, X_4_^2^, X_1_X_2_, X_2_X_3_, and X_2_X_4_, were eliminated, which indicated that the effects of extraction time (X_2_) and S/S ratio (X_4_) were not significant for the extraction of flavonoids from red and brown rice bran.

According to the analysis of variance, the models had good coefficient of determinations (*R*^2^) for Y_1_ (0.9670) and Y_2_ (0.9730) and were significant (*F*-value 0.0001, *p* < 0.05), which implied that the three factors (X_1_, X_3_, and X_4_) had an influence on the extraction efficiency of flavonoids. A higher *R*^2^ value indicates a better fit of the experimental model to the real data. On the other hand, a lower *R*^2^ value of *R*^2^ indicates worse correlation, but this can be used to elucidate the behavior of independent variables. The lack-of-fit test was not significant, which indicates an adequate fit of the models to the experimental data for all response variables.

### 2.4. Analysis of Response Surfaces

#### 2.4.1. Effect of Temperature on TFC

**Figure 5:**
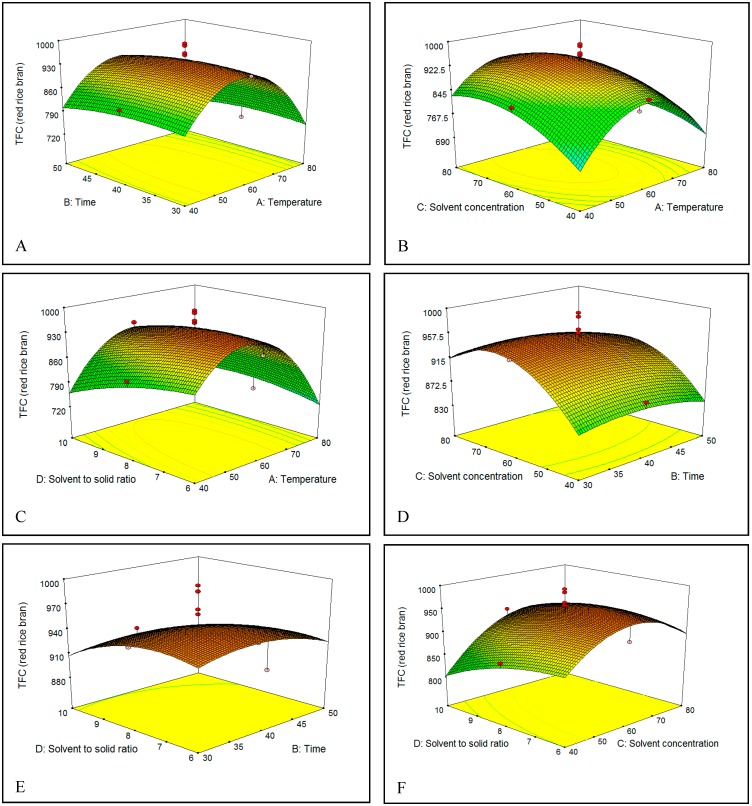
(**A**–**F**) Response surface plots for the effects of temperature, time, solvent percentage, and S/S ratio on total flavonoids content (TFC) of red rice bran.

**Figure 6:**
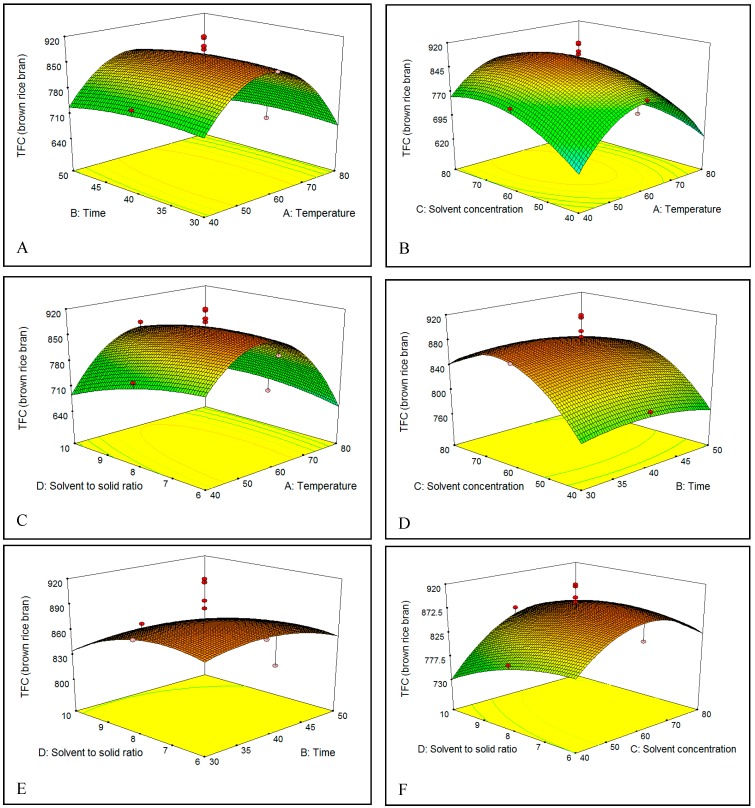
(**A**–**F**) Response surface plots for the effects of temperature, time, solvent percentage, and S/S ratio on total flavonoids content (TFC) of brown rice bran.

The effect of extraction temperature (40–80 °C) in ethanol solution on the TFC of brown and red rice bran is shown in Figure 5A–C and Figure 6A–C. On increasing the temperature from 40 to 60 °C, the TFC gradually increased in both brown and red rice bran extracts, which could be due to an increase in the rate of diffusion or the solubility of flavonoids in the solvent [33]. However, increasing the temperature to above 60 °C resulted in a decrease in the TFC of brown and red rice bran, which could be due to degradation by oxidation, or decomposition of the flavonoid structures in the extracts at high temperatures [30,36]. In industry, variations in the extraction temperature and the extraction time commonly occur because the temperature rise curves are easily affected by many factors.

#### 2.4.2. Effect of Extraction Time on TFC

Choosing an optimal extraction time for the solvent extraction process is an essential to minimize the energy cost of the process and inhibit the degradation of commercial compounds [37]. Thus, the influence of extraction time on the TFC was examined [Figure 5A,D,E and Figure 6A,D,E]. The extraction yield showed the same behavior for brown and red rice bran. In fact, no significant change in the TFC was observed when the extraction time increased from 30 to 50 min. The highest TFC was observed between 40 and 45 min. For the extraction of flavonoids and phenolics, some researchers have recommended short times [38,39], whereas others have recommended long times [40,41]. These differences in extraction time could be related to the sample type (seed, leaf, rhizome, or bark), particle size, type of solvent, and extraction method [37,42].

#### 2.4.3. Effect of Solvent Percentage on TFC

The concentration or percentage of solvent has been the most critical variable for extraction of phenolics from plants [38]. Figure 5B,D,F and Figure 6B,D,F show the relationship between the solvent percentage and the TFC. The TFC increased with increasing solvent percentage up to 72% (*v*/*v*) and decreased thereafter. The decrease in the TFC could be related to changes in flavonoid solubility in the ethanol/water mixture owing to changes in the polarity of the extractant. The dielectric constant of water is high, which causes the polarities of mixtures to vary with solvent percentage. This phenomenon might also be attributed to changes in the viscosity of the extractant, which would affect mass transfer. The viscosity of water is higher than that of ethanol, which enables water to penetrate the bran more completely and thereby increase recovery of polar and semipolar secondary metabolites [35]. Water always acts as a swelling agent in plants, whereas alcohols, such as ethanol, apparently interrupt bonding between the plant and matrix solutes, which can further affect the recovery [43]. Hence, a mixture of water and an organic solvent is more effective to extract secondary metabolites than water or organic solvents alone.

#### 2.4.4. Influence of S/S Ratio on TFC

Figure 5C,E,F and Figure 6C,E,F show that the initial increase of the S/S ratio from 6 to 8 mL/g increased the flavonoid recovery from bran of brown and red rice. This behavior could be related to an increase in the concentration gradient, which is the driving force for extraction.

Based on mass-transfer principles, the use of a higher S/S ratio results in a higher concentration gradient, which leads to enhanced diffusion and increases the extraction yield [31]. The same tendency was observed by Spigno [44] for phenol extraction from tea. However, in this study, the TFC yield decreased on increasing the S/S ratio from 8 to 10 mL/g. Similar results have been reported in a number of studies [45]. It may be that the high amount of solvent in the extraction system at higher S/S ratios does not affect the driving force for extraction because the mass-transfer process is mostly confined to the solid interior [46], which decreases the TFC.

Figure 5C,E,F and Figure 6C,E,F shows that an initial rise of the S/S ratio from 6 to 8 mL/g boosts the TFC recovery from bran of brown and red rice. This behavior could be related to the increase in the concentration gradient, which is the driving force in extraction.

### 2.5. Optimization and Prediction of Parameters

After applying a uniform design, which effectively narrowed the range of extraction conditions, some sophisticated tests were investigated in succession by orthogonal to obtain more efficient results. To maximize the TFC in the rice bran extract, the extraction conditions were optimized. Multiple regression analysis was used to achieve the optimal extraction conditions. The predicted models were used to optimize the extraction process based on the highest desirability values after regression analysis. The optimal variables for extraction of flavonoids from red and brown rice bran are shown in Table 3. The highest TFC value for red rice bran (958.14 mg QE/100 g DM) was predicted at a temperature of 58.5 °C, a solvent percentage of 71.5% (*v*/*v*), an extraction time of 36.2 min, and an S/S ratio of 7.94 mL/g. The highest TFC value for brown rice bran (782.52 mg QE/100 g DM) was predicted at a temperature of 56.7 °C, a solvent percentage of 74.4% (*v*/*v*), an extraction time of 36.9 min, and an S/S ratio of 7.18 mL/g.

### 2.6. Optimal Condition Validation

Experiments were performed under the optimized conditions to validate the models. As can be seen in Table 3, the experimental TFC values in both red and brown rice bran are similar to the predicted values, and there is no significant differences between the predicted and experimental TFC values. This result indicates that the developed individual models for the TFC were suitable for efficiently optimizing the extraction conditions. Thus, the present method is accurate, reliable, and reproducible.

### 2.7. Phytochemical Properties

The amounts of major flavonoids in the extracts of red and brown rice bran are displayed in Table 4. Five flavonoids were detected in the extracts, namely, quercetin, apigenin, catechin, luteolin, and myricetin. Red rice bran exhibited a higher content of individual flavonoids than brown rice bran, and apigenin was the predominant flavonoid in the active extracts of both rice brans. Significant differences were observed between red and brown rice bran for all identified flavonoid compounds. Five phenolic acids, namely, protocatechuic acid, syringic acid, ferulic acid, cinnamic acid, and *p*-coumaric acid, were identified in the extracts of red and brown rice bran. As shown in Table 4, among the identified phenolic acids, *p*-coumaric acid was the predominant phenolic acid in the active extract of both red and brown rice bran. With the exception of ferulic acid and *p*-coumaric acid, the contents of all phenolic acids were significantly different in red and brown rice bran, with no cinnamic acid detected in brown rice bran. The extract of red rice bran had a higher content of individual flavonoids and phenolic acids than brown rice bran.

### 2.8. Antioxidant Activities

#### 2.8.1. Nitric Oxide (NO) Scavenging Activity

**Figure 7:**
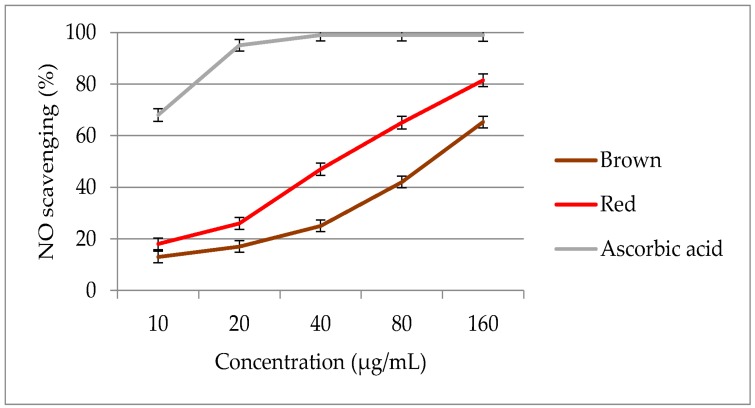
Nitric oxide scavenging activity of optimized extracts of red and brown rice bran.

The optimized extracts from red and brown rice bran were examined for NO scavenging activity at various concentrations (Figure 7). The extracts were tested in the concentration range of 10–160 μg/mL. The NO activities of the pigmented rice brans ranged from 13.0 to 81.2%, with significant differences (*p* < 0.05) among the pigmented rice genotypes. A significant increase (*p* < 0.05) in NO activity was observed on increasing the rice bran concentration from 10 to 160 μg/mL. The extract of brown rice bran demonstrated a weaker NO scavenging activity than the extract of red rice bran.

In this study, the optimized extract of red rice bran exhibited a lower EC_50_ value (41.3 μg/mL) than the extract of brown rice bran (118 μg/mL), indicating that the free radical scavenging power of red rice br.an is h.igh.er than that of the brown rice genotype. The EC_50_ values of the extracts of red and brown rice bran were lower than that of ascorbic acid (4.68 μg/mL). When comparing the antioxidant activities of different extracts, extracts with low EC_50_ values act as stronger free radical inhibitors [47].

The results of recent studies have shown that red and black rice bran have potent free radical scavenging power as compared to light brown rice bran [8,48]. Rice bran, though small in amount, is rich in phytochemicals and antioxidant agents; hence, removal of the bran layer during the production of polished rice power decreases the antioxidant activity [49].

The NO scavenging activity results suggest that bran of red rice could be a valuable source of antioxidants as compared to brown rice, which is more frequently consumed. Globally, people now eat different pigmented rice genotypes, such as black, red, brown, and purple, which have been developed as healthy foods. Moreover, the food industry is currently interested in increasing the use of natural antioxidants in primary or basic raw materials because of safety concerns over the use of synthetic antioxidants [50]. Red rice bran, as a rich source of natural antioxidants, has considerable potential for applications in the food and pharmaceutical industries.

#### 2.8.2. 2,2-Diphenyl-2-picrylhydrazyl (DPPH) Scavenging Activity

**Figure 8:**
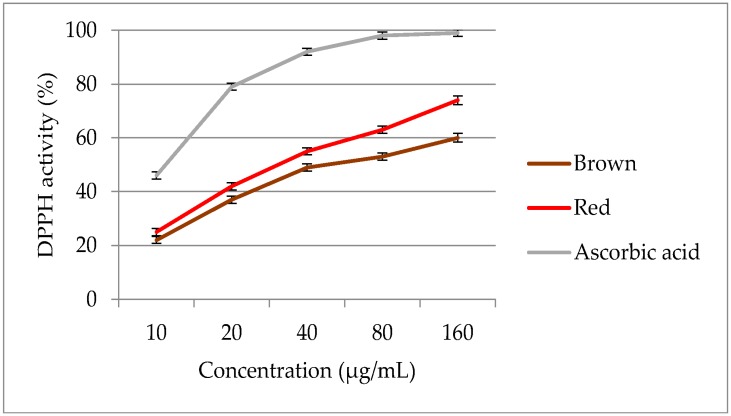
DPPH scavenging activity of optimized extracts of red and brown rice bran.

On increasing the extract concentration from 10 to 160 µg/mL, the DPPH radical scavenging power increased significantly (Figure 8). The DPPH activity of red and brown rice bran ranged from 12.4 to 74.9%, and significant (*p* < 0.05) differences in DPPH activity were observed between the two pigmented rice varieties. In terms of DPPH activity, the extract of red rice bran exhibited the higher activity than the extracts of brown rice bran. Both pigmented rice brans exhibited lower DPPH activities than ascorbic acid (EC_50_ = 11.74 μg/mL). Red rice bran had a lower EC_50_ value (33.6 μg/mL) than brown rice bran (48.7 μg/mL), indicating the potent antioxidant properties of red rice bran. In the extract of red rice bran, a high DPPH activity with a low EC_50_ value could be related to a greater concentration of flavonoids in this genotype. Thus, red rice bran extracts may provide superior radical scavenging activities owing to a higher amount of phytocompounds. The results of several studies have indicated that the free radical scavenging power of pigmented rice brans show significant correlation with the content and type of phytochemicals [51,52,53]. After collecting more data, especially data from industry, the models should be reevaluated. Subsequently, the design space should also be recalculated to realize a more reliable and flexible pharmaceutical process.

## 3. Materials and Methods

### 3.1. Rice Samples

Rice grains, which were harvested in the summer of 2015, were obtained from the Rice Research Institute of Iran (RRII), Rasht, Iran. The cultivated rice included two different pigmented rice genotypes with a light brown or red pericarp color (red: IR5009; light brown: IR226b). After harvest, all rice grains were dried in an oven at 39 ± 1 °C until the moisture content decreased to 1.3 ± 1%. A rice dehusker was used for de-hulling and a rice miller (8% degree) was used for polishing to obtain the milled rice bran. The milled samples were sieved using sieve of 1.80 μm. Before extraction in order to inactivate endogenous lipases, rice bran was heated at 100 °C for 15 min. After cooling of samples rice bran was extracted two times with 4 mL of 100% hexane for 2 h, at room temperature. After evaporating the residual hexane under reduced pressure, the defatted bran was extracted.

### 3.2. Preliminary Study of Extraction Parameters

To find the appropriate range of variables for the extraction process, preliminary experiments were conducted. Extraction was done using digital electric heating thermostat circulating water bath with double line six hole (HH.S21-6-S, China, 1500 W). Temperature during extraction adjusted with Temperature control panel. Extraction times of 10–60 min, extraction temperatures of 20–80 °C, S/S ratios of 2–12 mL/g, and ethanol or acetone percentages of 0–100% (*v*/*v*) were chosen as variables for the extraction process. A one-factor-at-a-time method was used to investigate the influence of each factor on the targeted yield in extracts. Experiments were carried out in triplicate to ensure reproducibility.

### 3.3. RSM

RSM is an experimental statistical technique applied for multiple regression analysis using quantitative data obtained from properly designed experiments [54]. For the efficient extraction of active compounds, including phenolic acids and flavonoids, from pigmented rice bran, various parameters that influenced the extraction efficiency were optimized. In this study, to obtain the optimal extraction conditions, the relationships among temperature (X_1_), time (X_2_), solvent percentage (X_3_), and S/S ratio (X_4_) were investigated using CCD. The quadratic polynomial step-by-step regression method and data were analyzed using Design-Expert (version 7, Stat-Ease, Inc., Minneapolis, MN, USA) software. The model given below was used to predict the response variables.

*Y* = *b*_0_ + *b*_1_X_1_ + *b*_2_X_2_ + *b*_3_X_3_ + *b*_1_^2^X_1_^2^ + *b*_2_^2^X_2_^2^+ *b*_2_^3^X_2_^3^ + *b*_1_*b*_2_X_1_X_2_ + *b*_1_*b*_3_X_1_X_3_ + *b*_2_*b*_3_X_2_X_3_,

where *Y* is the predicted dependent variable; *b*_0_ is a constant that fixes the response at the central point of the experiment; *b*_1_, *b*_2_, and *b*_3_ are the regression coefficients for the linear effect terms; *b*_1_*b*_2_, *b*_1_*b*_3_, and *b*_2_*b*_3_ are the interaction effect terms; and *b*_1_^2^, *b*_2_^2^, and *b*_3_^2^ are the quadratic effect terms. The regression coefficients of individual linear, quadratic, and interaction terms were determined according to analysis of variance (ANOVA). To visualize the relationship between the response and experimental levels of each factor, and to deduce the optimal conditions, the regression coefficients were used to generate 3-D surface plots and contour plots fr.om the fitted polynomial equation. The factor levels we.re coded as −1 (low), 0 (central point or middle), and +1 (high). The variables were coded according to the following equation:

$$Xi=\frac{X_{i}-X_{0}}{\Delta X}\;$$

where X*i* is the (dimensionless) coded value of the variable X*_i_*, X_0_ is the value of X at the central point, and ΔX is the step change.

### 3.4. TFC Analysis

The pigmented rice extract (500 μL) was incubated for approximately 6 min with distilled water (2 mL). Subsequently, 5% sodium nitrite (150 μL), 10% aluminum chloride (150 μL), and 4% sodium hydroxide (2 mL) were added at room temperature, and the volume of the test tube was made up to 5 mL with distilled water. The incubated (15 min) sample, which had a pink color, was read at 510 nm against a blank reagent containing plant extract (500 μL) and a few drops of acetic acid diluted with methanol (3 mL). The flavonoid content in the extract was estimated using a standard curve for rutin hydrate in methanol under the same conditions [55]. The results were expressed in mg QE/100 g DM.

### 3.5. HPLC Analysis of Extracts

Qualitative and quantitative analyses of the samples were performed using an Agilent HPLC 1200 system (Agilent Technologies, Santa Clara, CA, USA) coupled to an API 3200 triple quadrupole mass spectrometer (Agilent Corporation, Santa Clara, CA, USA). An Agilent Eclipse Plus C18 column (1.8 μm, 4.6 mm × 150 mm) was equipped with an Agilent C18 guard column (5 μm, 2.1 mm × 12.5 mm). The mobile phase consisted of acetonitrile (solvent A) and 0.05% aqueous formic acid (solvent B). Polyphenols separated and eluted using the following gradient: at 0.01 min 85% B, at 3 min 81% B, at 9 min, 80% B, at 12 min 70% B, at 12.5 min, 52% B, at 14 min, 48% B, at 17 min, 46% B; at 18.5 min, 40% B; at 20 min, 19% B. Post running time was 5 min and the column temperature was 35 °C. In addition, the injection volume and flow rate were 5 μL and 1 mL/min, respectively. For each sample three injections were performed. 

### 3.6. Evaluation of Antioxidant Activity

#### 3.6.1. DPPH Assay

The rice bran extracts were examined for their hydrogen-donating ability towards DPPH, which is a stable free radical. The sample extracts and ascorbic acid were adjusted to 100 μL with methanol, mixed with 3 mL of 0.1 mM DPPH in methanol, and then vortexed well. The solutions were incubated in the dark for 30 min. The scavenging activities of the extracts were determined from the absorbance at 517 nm against methanol as a blank solution, and the results were expressed as the concentration of a sample that induces a response halfway between the baseline and the maximum after a specified exposure time (EC_50_) [56]. The following formula was used to calculate the scavenging activity:

% inhibition = [(absorbance_control_ − absorbance_sample_)/absorbance_control_)] × 100.

#### 3.6.2. NO Scavenging Activity

Various concentrations (50–250 μg/mL) of rice bran extracts were prepared with a total volume of 3 mL. A 1.0 mM solution of sodium nitroprusside in phosphate buffer (0.5 M, pH 7.4) was prepared. Subsequently, 2 mL of this mixture was mixed with the rice bran extracts, which were then shaken well and incubated at 37 °C for 6.0 min. During incubation, the Griess reagent was prepared freshly by adding 0.1% α-naphthyl-ethylenediamine in water to 1% sulfuric acid in 5% *ortho*-phosphoric acid. The Griess reagent (2.5 mL) was mixed with the incubated solution, which when then shaken well using a vortex mixer. A spectrophotometer was used to record the absorbance of the extracts at 540 nm. Ascorbic ac.id was used as a positive control [57].

## 4. Conclusions

In this study, the optical extraction conditions were determined to obtain high yields of secondary metabolites (flavonoids) from the bran of two pigmented (red and brown) rice genotypes by combining CCD with RSM. Ethanol was found to be a more favorable solvent than acetone for flavonoid extraction. The optimal temperature, solvent percentage, extraction time, and S/S ratio to maximize the TFC value of red rice bran (958.14 mg QE/100 g DM) were 58.5 °C, 71.5% (*v*/*v*), 36.2 min, and 7.94 mL/g, respectively, whereas the maximum TFC value of brown rice bran (782.52 mg QE/100 g DM) was predicted at 56.7 °C, 74.4% (*v*/*v*), 36.9 min, and 7.18 mL/g, respectively. The extract of red rice bran exhibited higher concentrations of individual flavonoid compounds than the extract of brown rice bran. Moreover, the free radical scavenging power of the red rice bran extract obtained under optimal conditions was higher than that of the brown rice bran extract. Hence the optimum extraction conditions obtained in this study could be used as a standard or base line information for industrial processing of these rice varieties. Therefore, we consider these results to provide new information for further in vitro/in vivo biological studies (e.g., antiproliferative activity, anti-inflammatory activity) on these pigmented rice varieties. Optimized extracts (secondary metabolite rich extracts) of these pigmented rice varieties might be used as a natural antioxidants and natural colorants in food products (e.g., sausage, snakes). Future experimental investigations are needed to estimate the stability of secondary metabolites in the final products.

## Figures and Tables

**Table 1:** Analysis of variance for the experimental results of total flavonoid content from red rice bran.

Parameter	Degree of Freedom	Sum of Squares	*F*-Value	Prob > *F*
Model Intercept	14	31,677.40	27.10	0.0001
Linear				
X_1_	1	21,868.40	18.71	0.0006
X_2_	1	1573.64	1.35	0.2645
X_3_	1	15,254.20	13.05	0.0026
X_4_	1	5896.98	5.05	0.0482
Quadratic				
$X_{1}^{2}$	1	60,251.40	51.55	0.0001
$X_{2}^{2}$	1	555.73	0.48	0.5017
$X_{3}^{2}$	1	8865.41	7.59	0.0148
$X_{4}^{2}$	1	829.75	0.71	0.4127
Interaction				
X_1_X_2_	1	34.81	0.03	0.8653
X_1_X_3_	1	14,604.70	12.5	0.003
X_1_X_4_	1	15,500.30	13.26	0.0024
X_2_X_3_	1	278.89	0.24	0.6323
X_2_X_4_	1	186.32	0.16	0.6953
X_3_X_4_	1	5461.21	4.67	0.0472
Lack of fit	10	1153.44	0.96	0.5543
Pure error	5	1199.45		
Residual	15	1168.77		
*R*^2^ Adjusted			0.926	
*R* ^2^			0.967	
C.V.%			4.284	
Cor. Total	29			

**Table 2:** Analysis of variance for the experimental results of total flavonoid content from brown rice bran.

Parameter	Degree of Freedom	Sum of Squares	*F*-Value	Prob > *F*
Model Intercept	14	31,807.50	27.24	0.0001
Linear				
X_1_	1	21,362.70	18.28	0.0006
X_2_	1	1592.80	1.33	0.2641
X_3_	1	15,743.53	12.60	0.0023
X_4_	1	5903.30	5.16	0.0402
Quadratic				
$X_{1}^{2}$	1	61,044.2	52.27	0.0001
$X_{2}^{2}$	1	502.80	0.43	0.5217
$X_{3}^{2}$	1	8768.68	7.51	0.0152
$X_{4}^{2}$	1	884.88	0.76	0.3977
Interaction				
X_1_X_2_	1	44.46	0.038	0.8479
X_1_X_3_	1	15,441.20	13.22	0.0024
X_1_X_4_	1	15,226.90	13.04	0.0026
X_2_X_3_	1	212.65	0.18	0.6756
X_2_X_4_	1	180.97	0.15	0.6994
X_3_X_4_	1	5798.44	4.97	0.0416
Lack of fit	10	1145.77	0.95	0.5626
Pure error	5	1211.76		
Residual	15	1167.77		
*R*^2^ Adjusted			0.929	
*R* ^2^			0.973	
C.V.%			4.701	
Cor. Total	29			

**Table 3:** Predicted and experimental values of TFC obtained under the optimal extraction conditions.

Rice Bran	Temperature (°C)	Time (min)	Solvent Percentage (%)	S/S Ratio (mL/g)	Desirability	TFC (mg QE/100 g DM)	
						Predicted	Experimental
Red	58.5	36.2	71.5	7.94	0.964	958.14	962.38
Brown	56.7	36.9	74.4	7.18	0.971	782.52	788.21

**Table 4:** Identified flavonoid and phenolic acid compounds from an optimized extract of red and brown rice bran with information of retention time, wavelength, and linear regression parameters.

Flavonoids and Phenolic Acids	Rice Bran		t_r_ (min)	λ (nm)	Calibration Equation *	*R* ^2^	LOD	LOQ
	Red	Brown						
Catechin	4.26 ± 0.34 ^a^	2.14 ± 0.16 ^b^	3.82 ± 0.02	280	(0.138 ± 0.005)x + (−0.086 ± 0.040)	0.993	0.55	1.68
Quercetin	5.16 ± 0.48 ^a^	1.65 ± 0.10 ^b^	9.52 ± 0.02	360	(0.085 ± 0.001)x + (−0.112 ± 0.021)	0.992	1.14	3.52
Myricetin	7.55 ± 0.45 ^a^	4.41 ± 0.24 ^b^	11.47 ± 0.01	360	(0.060 ± 0.001)x + (−0.086 ± 0.036)	0.994	0.82	2.51
Luteolin	6.47 ± 0.27 ^a^	1.08 ± 0.10 ^b^	14.65 ± 0.02	360	(0.147 ± 0.002)x + (−0.101 ± 0.005)	0.995	1.09	3.29
Apigenin	11.63 ± 0.69 ^a^	7.69 ± 0.51 ^b^	18.04 ± 0.03	360	(0.029 ± 0.001)x + (−0.041 ± 0.015)	0.986	1.10	3.32
Cinnamic acid	6.03 ± 0.38 ^a^	ND	10.22 ± 0.02	320	(0.158 ± 0.004)x + (−0.123 ± 0.073)	0.993	0.93	2.84
Syringic acid	6.23 ± 0.37 ^a^	7.15 ± 0.36 ^b^	15.60 ± 0.03	280	(0.017 ± 0.001)x + (−0.044 ± 0.005)	0.990	1.18	3.62
*p*-coumaric acid	8.16 ± 0.49 ^a^	7.94 ± 0.53 ^a^	15.83 ± 0.03	320	(0.251 ± 0.005)x + (−0.117 ± 0.004)	0.995	1.16	3.54
Ferulic acid	5.56 ± 0.26 ^a^	5.12 ± 0.44 ^a^	17.04 ± 0.01	320	(0.055 ± 0.004)x + (−0.096 ± 0.028)	0.991	0.91	2.75
Protocatechuic acid	2.04 ± 0.11 ^a^	1.60 ± 0.12 ^b^	18.01 ± 0.03	320	(0.068 ± 0.007)x + (−0.054 ± 0.064)	0.998	1.10	3.37

^a, b^ Data were the mean ± standard deviation of triplicate measurements. Different superscript lower case letters in each row indicated significant difference at *p* < 0.05 (Duncan’s test). ND: not detected; *: *n* = 3; t_r_: retention time; λ: wavelength; LOD: limit of detection; LOQ: limit of quantification.
